# How Perceived Control and Task Value Relate to Achievement Emotions in Academic Study Settings

**DOI:** 10.3390/bs16050791

**Published:** 2026-05-16

**Authors:** Daniela Raccanello, Giada Vicentini, David W. Putwain

**Affiliations:** 1 Department of Human Sciences, University of Verona, 37129 Verona, Italy; giada.vicentini@univr.it; 2 School of Education, Liverpool John Moores University, Liverpool L1 9DE, UK; d.w.putwain@ljmu.ac.uk

**Keywords:** control–value theory, achievement emotions, perceived control, task value, university students

## Abstract

Control–value theory (CVT) is a robust framework for understanding the antecedents of achievement emotions. However, few studies examined the interaction between control and value appraisals across a broad range of emotions. The aim of this study was to explore the interactive role of control and value in predicting 10 achievement emotions—three positive activating emotions (enjoyment, pride, and hope), two positive deactivating emotions (relief and relaxation), three negative activating emotions (anxiety, anger, and shame), and two negative deactivating emotions (hopelessness and boredom)—felt in relation to the academic setting of studying. We recruited 166 and 126 Italian university students attending two consecutive psychological modules (General Psychology and Developmental and Educational Psychology) within the same degree programme. Data were collected via self-report questionnaires, with partial overlap between samples, as a substantial proportion of students participated in both modules. Through regression analyses, control and value showed the expected pattern of associations, being positively related to positive emotions and negatively related to negative emotions. Evidence for interaction effects was limited across emotions and modules: significant interactions emerged for anger and shame in the first module and for enjoyment and relaxation in the second, although these effects were generally small in magnitude. Overall, the findings provide partial support for CVT assumptions, suggesting that value may moderate the relation between control and achievement emotions in specific contexts, but not in a consistent or uniform way across emotional outcomes.

## 1. Introduction

When confronted with learning-related tasks, students can experience a range of emotions arising from interactions among various factors, which, in turn, affect their performance through cognitive, metacognitive, and motivational mechanisms (Pekrun et al., 2023). At the same time, emotions are relevant in their own right, as they are intrinsically linked to students’ well-being, one of the key goals of educational institutions. While in the past researchers mainly focused on test anxiety (Zeidner, 2007) and a few other emotions related to attributional processes such as pride and shame (Weiner, 2018), it is now widely recognised that many different emotions play a pivotal role in learning contexts. Accordingly, it becomes important to examine a broad range of achievement emotions and their appraisals in order to identify ways to promote adaptive emotions for both students’ learning and well-being.

In the last two decades, Pekrun’s control–value theory (CVT) of achievement emotions (Pekrun, 2006, 2024, 2025; Pekrun et al., 2023) has given a great impetus to research. According to this approach, control and value appraisals are recognised as the main proximal individual antecedents of achievement emotions, in line with literature documenting the relevance of appraisals for the emotion generation process (Moors et al., 2013). Notwithstanding the number of studies about the nature of the relation between emotions and their antecedents as postulated by CVT (Pekrun et al., 2023), only rarely has attention been paid to examining how control and value interact. In the few studies about this, researchers focused on a small number of emotions (Bieg et al., 2013; Goetz et al., 2010; Lauermann et al., 2017; Putwain et al., 2018, 2021). Only one study, to date, has taken into account a more extensive repertoire of emotions (Shao et al., 2020). Nonetheless, Shao et al. (2020) did not consider deactivating positive emotions such as relief and relaxation.

These two emotions have been understudied in relation to their appraisals (Graham et al., 2023; Richardson et al., 2016). They are nevertheless important because they are frequently experienced at different school and academic stages (Raccanello et al., 2013, 2018b, 2022a, 2022b; Raccanello & Hall, 2021). They are associated with positive learning outcomes (see the following paragraph), similarly to positive activating emotions, but they also have peculiar characteristics differentiating them from those emotion types.

Relief seems to occur in two situations (Graham et al., 2023). People report feeling relieved either when they realise they have avoided a potentially negative experience or when a negative experience has ended. In the first case a counterfactual thinking is at play (Markman et al., 1993) while in the second the appraisals of relief are simply based on a temporal criterion. In learning contexts, both situations can be very frequent and be related to a variety of antecedents. Therefore, extending knowledge of the nature of the appraisals underlying relief can be highly relevant to further understand how students feel and, in turn, how to support them.

Relaxation or calm is conceptualised as a highly pleasant and low-activating emotion (Richardson et al., 2016) and could be considered an antonym of anxiety, which has been largely investigated in relation to achievement (Zeidner, 2007). However, relaxation has been disregarded by research on learning. It differentiates from enjoyment, usually associated with a driving, proactive force leading people towards resources and rewards, for its function of “turning off” an individual’s attention to restore one’s own energy (Kawabata & Mallett, 2022; Richardson et al., 2016). Therefore, in some learning settings, relaxation could be a more adequate emotion compared to enjoyment. For example, in some contexts expressing a highly activating emotion such as enjoyment could be considered inadequate, e.g., when studying in a place where other students need a quiet environment and not to be disturbed by people who externalise a high enthusiasm. Further knowledge on how control and value interact across a broad range of emotions—and particularly regarding the under-investigated deactivating positive emotions of relief and relaxation—is therefore needed as a central resource for developing ways to intervene on emotions’ appraisals, in order to foster adaptive achievement emotions and reduce those associated with more negative processes.

Despite the relevance of CVT (Pekrun, 2006, 2024, 2025; Pekrun et al., 2023), research on the interaction between control and value in predicting achievement emotions remains limited and fragmented. In particular, previous studies are characterised by several gaps: (a) they provide mixed and still limited evidence on the interactive (multiplicative) role of control and value; (b) they typically focus on a restricted set of achievement emotions, often neglecting the full range of emotions differing in valence and activation; and (c) they are often conducted in learning contexts that are not highly relevant to students’ academic interests, leaving open questions about how these appraisals operate in more personally meaningful settings. To address these gaps, we recruited Italian university students at the beginning of their first and second year of a Bachelor’s degree course in Psychological Sciences, considering CVT as the main theoretical framework. Unlike previous research (e.g., Shao et al., 2020), which examined control and value interactions in a foreign language course unrelated to students’ major, we focused on two compulsory psychology modules (General Psychology, GP, and Developmental and Educational Psychology, DEP). Given that the choice of a university major such as Psychology is often aligned with students’ interests, these contexts were likely associated with relatively high task value, making them particularly suitable for examining the joint role of control and value in shaping achievement emotions. Furthermore, we extended prior research by considering a broader set of achievement emotions (*N* = 10), differentiated by valence and activation. To our knowledge, no previous study has examined the interaction between control and value across such a wide range of emotions, including positive deactivating emotions such as relief and relaxation.

### 1.1. The Control–Value Theory

According to CVT (Pekrun, 2006, 2024, 2025; Pekrun et al., 2023) and in line with previous literature, emotions are multi-component phenomena that encompass affective, cognitive, subjective, physiological, and expressive-behavioural components, arising as individuals’ responses to something relevant for the self. For example, a student strongly motivated for her university choice and thinking that she has long waited for increasing knowledge about how people function, can approach the first book she has to study pleasantly (affective component), happy for understanding the contents (cognitive component), energised (physiological component), searching time for studying while neglecting leisure activities (motivational component), and smiling (expressive-behavioural component). At the same time, another student who feels anxiety when studying could open the same book nervously (affective component), worrying about failure at the exam (cognitive component), sweating (physiological component), trying to excogitate excuses for closing the book as soon as possible (motivational component), and transmitting his negative emotion through facial expressions and gestures (expressive-behavioural component).

In both cases, the emotion can be thought of as an achievement emotion. Consistent with previous literature (Pekrun, 2006, 2024, 2025; Pekrun et al., 2023), we define achievement emotions as those emotions pertaining to learning activities or outcomes, differentiated for at least two underlying dimensions, namely valence and activation. While the first accounts for the pleasantness of the affective state, activation concerns physiological arousal. The combination of the two dimensions gives rise to four quadrants (as represented in Table 1)—positive activating emotions such as enjoyment, pride, and hope; positive deactivating emotions such as relief and relaxation; negative activating emotions such as anxiety, anger, and shame; and negative deactivating emotions such as hopelessness and boredom.

According to the CVT, we can resort to a third dimension for classifying emotion, i.e., object focus (Pekrun, 2006, 2024, 2025; Pekrun et al., 2023). Object type includes the reference to activities or outcomes (Pekrun et al., 2023): emotions felt while studying are probably activity-focused, while those related to an exam are probably outcome-focused. Moreover, the object can be characterised by different temporal relations, i.e., present-focused (in this case, emotions are concurrent, as it is possible while studying), future-focused (being prospective), or past-focused (being retrospective). Asking students to report how they feel when studying could be a way to operationalise emotions as focused concurrently on a specific activity. However, while we could presume that all the emotions measured in this way have these characteristics, we cannot determine if pride, relief, and shame (typically retrospective emotions) or hope, anxiety, and hopelessness (typically prospective emotions) are referred to the studying activity (i.e., focusing on different sub-phases of it) or rather to situations preceding or following the activity itself.

According to the CVT (Pekrun, 2006, 2024, 2025; Pekrun et al., 2023), achievement emotions can be described considering their intertwinement with a range of antecedents and outcomes. Control and value appraisals are the most relevant antecedents, impacting directly on achievement emotions. These antecedents, in turn, are affected by more distal factors relating to the individual (e.g., personality traits) and the situation (e.g., perceptions of the learning context, such as task clarity and difficulty, or teachers’ enthusiasm). Achievement emotions then impact performance through the mediation of cognitive, metacognitive, and motivational factors, such as strategy use, effort, and self-regulation (Pekrun et al., 2023). Notwithstanding some exceptions, data usually support that positive emotions are associated with better performance and vice versa for negative emotions; however, the findings about the role of positive deactivating emotions and negative activating emotions are sometimes inconsistent (Loderer et al., 2020; Pekrun, 2006, 2024, 2025; Pekrun et al., 2023; Shao et al., 2020). All these relations are assumed to be reciprocal, with recursive links. Finally, a key CVT assumption is the domain-specificity of achievement emotions, which are organised differently across contexts (Pekrun, 2006, 2024).

### 1.2. Control and Value

As anticipated, the most relevant determinants of achievement emotions are control and value appraisals (Pekrun, 2006, 2024, 2025; Pekrun et al., 2023). Perceived control (henceforth control) refers to the appraisal of control concerning actions and outcomes. Feeling in control of one’s achievement is a source of positive emotions, activating (e.g., for enjoyment, pride, and hope) and in some cases deactivating (e.g., for relaxation, for example, but not for relief), also among university students. Typically, the effect is opposite for feeling a lack of control, which is generally associated with increased negative activating emotions, such as anxiety, anger, and shame, and also deactivating emotions, such as hopelessness (Pekrun et al., 2023). For the negative deactivating emotion of boredom, however, CVT assumptions suggest that increases in it would be associated with both low and high control (Pekrun et al., 2023). Nevertheless, a curvilinear relation between boredom and control appears less likely among university students (Shao et al., 2020). The challenges posed by learning and attainment tasks should make it unlikely to feel bored due to excessive competence. As for the conceptual relations of control with similar constructs, we specify that self-efficacy is one facet of control (the other being causal attribution) and that academic self-concept underpins both self-efficacy and control (Pekrun, 2006).

Task value (henceforth value) refers to the subjective perception of the relevance of a task. Following Eccles and Wigfield (2023), it can include the so-called intrinsic value (i.e., when the task triggers interest or curiosity) and forms of extrinsic value like attainment value (i.e., when the task is relevant for self-defining issues, such as getting good grades) and utility value (i.e., when the task is important for short- or long-term objectives such as for other modules or for a future job). Intrinsic value has been found to be positively related to positive activating emotions such as enjoyment and positive deactivating emotions such as relaxation and negatively to negative activating emotions such as anger and negative deactivating emotions such as boredom for psychology university students (Pekrun et al., 2023).

Therefore, the CVT indicates the beneficial or detrimental independent effects of control and value generally on positive activating and deactivating emotions and on negative activating and deactivating emotions, respectively. Nevertheless, although CVT allows for the possibility that the two appraisals may combine their effects in multiplicative ways (Pekrun, 2006, 2024), empirical support for this interactive pattern is still mixed (e.g., Putwain et al., 2018; Shao et al., 2020, as described later). This perspective is in line with classical appraisal theories recognising the simultaneous presence and possible links of more than one appraisal (Arnold, 1960). For example, a student is assumed to get more angry or ashamed—for a variety of individual or contextual reasons, such as perceiving difficulties in understanding or assessing one’s own learning strategies as poor—while studying something which is deemed as personally relevant rather than something considered not important for the self; similarly, another could enjoy more while studying contents perceived as relevant for their future, compared to another student perceiving no utility in mastering them. To sum up, it has been suggested that all forms of value may amplify the impact of control on emotions. However, both appraisals should be necessary for generating emotion (Shao et al., 2020). In other terms, for positive activating and deactivating emotions, higher control would increase their intensity, and the lack of control would increase negative activating and deactivating emotions (even if it could not always be the case for boredom), while value may moderate these relations—although this pattern has not been consistently observed across studies.

Notwithstanding CVT assumptions about the nature of the relation between control and value, their interaction has rarely been tested in the literature (for exceptions see Bieg et al., 2013; Goetz et al., 2010; Lauermann et al., 2017; Putwain et al., 2018, 2021; Shao et al., 2020) and never with a repertoire of emotions corresponding to all four quadrants of the valence-activation taxonomy (neglecting positive deactivating emotions) and for a domain deemed as a core one for a university course.

As exceptions, some studies examined this issue with secondary school students, but with a reduced number of emotions (Bieg et al., 2013; Lauermann et al., 2017; Putwain et al., 2018, 2021). Bieg et al. (2013) involved eighth and eleventh graders and measured control and value (operationalised as attainment value) with a single item, focusing on four subjects (mathematics, physics, German, and English) in lesson-related settings. They observed that state and trait pride, anxiety, and boredom were predicted by control, value, and their interaction, consistent with the CVT, with a positive interaction for pride and negative interactions for anxiety and boredom. Lauermann et al. (2017) revealed that high intrinsic and utility value amplified the negative relation between control and worry for third, fourth, and sixth graders in mathematics and reading. Putwain et al. (2018, 2021) examined whether different intrinsic, attainment, and utility values had differential outcomes for mathematics in the lesson setting. Involving sixth graders longitudinally (Putwain et al., 2018), they found that the positive relation between control and enjoyment was amplified by attainment value, while the positive relation between control and boredom was reduced by intrinsic value. With fifth graders, Putwain et al. (2021) also found that control and intrinsic value interacted in predicting enjoyment, with a stronger positive relation between control and this emotion at high value.

Only two studies examined the interaction between control and value involving university students (Goetz et al., 2010; Shao et al., 2020). Goetz et al. (2010) investigated control and intrinsic value (through one item) about everyday situations for three emotions, namely enjoyment, pride, and contentment. In both achievement (studying, attending lessons) and non-achievement settings (watching television, having lunch), they revealed positive interactions for each emotion. Shao et al. (2020) involved university students, assessing a larger variety of emotions and using a composite measure of intrinsic, attainment, and utility value for the learning setting in a foreign language domain (English for Chinese students). They detected significant positive interactions between control and value for enjoyment, pride, and hope, and negative interactions for anxiety, anger, shame, hopelessness, and boredom. The relations between control and positive emotions and those between control and negative emotions were stronger when the value was high.

On the whole, the few studies examining the interaction between control and value only partially confirmed the CVT assumptions about their multiplicative effects: while higher control and value would increase positive emotions, and the lack of control and value would increase negative emotions, findings about the moderating role of value are still mixed. Moreover, to our knowledge, no previous study focused on subjects particularly relevant for students’ university courses, on the interaction between control and value in relation to a large number of positive and negative, activating and deactivating achievement emotions, and in particular no one focused on positive deactivating emotions with about the same sample of students in two consecutive years.

### 1.3. Aims

We included emotions from the four quadrants of Pekrun’s (2006, 2024, 2025) taxonomy (comprising also positive deactivating emotions, currently understudied, particularly in academic contexts); we examined broadly the same sample of university students in two consecutive years; and we operationalised domain-specificity of emotions focusing on the learning setting for two subjects deemed major for the university course. The main aim was to investigate how control, value, and their interaction related to 10 achievement emotions, differentiated as positive activating (enjoyment, pride, and hope), positive deactivating (relief and relaxation), negative activating (anxiety, anger, and shame), and negative deactivating (hopelessness and boredom).

We choose these emotions based on Kleine et al. (2005), including at least two emotions for each quadrant. They can also be distinguished according to their object focus: enjoyment, relaxation, anger, and boredom as activity-focused emotions; hope, anxiety, and hopelessness as prospective outcome emotions; and pride, relief, and shame as retrospective outcome emotions (Pekrun et al., 2023). We considered the academic setting of study for two modules, GP and DEP. A module (referred to as a “course” in many university/higher education systems) is a discrete component of a degree programme taught and assessed over one or two semesters. We formulated the following hypotheses.

**H1.**  *Control and value will be positively associated with positive activating and deactivating emotions and negatively associated with negative activating and deactivating emotions.*

**H2.**  *In line with the CVT framework, we examined whether value moderates the relation between control and achievement emotions. Given that previous findings on the interactive (multiplicative) role of control and value are mixed, this hypothesis is considered exploratory. Specifically:*

**H2a.**  *Value will strengthen the positive association between control and positive activating and deactivating emotions (i.e., the positive relation between control and these emotions will be stronger at higher levels of value).*

**H2b.**  *Value will strengthen the negative association between control and negative activating and deactivating emotions (i.e., the negative relation between control and these emotions will be stronger at higher levels of value).*

As a supplementary analysis, we also examined whether students perceived the two modules as valuable activities through an open-ended question on the reasons for their module-related emotions (see Supplementary Materials, SM, Paragraph S1).

## 2. Materials and Methods

### 2.1. Participants, Research Design, and Procedure

#### 2.1.1. Participants

We asked students enrolled in a bachelor’s degree course in psychological sciences at a north eastern Italian university (the number of students for each year is 225) to participate in a survey on two modules in two consecutive years. The final sample included 166 students (*M_age_* = 21.20; *SD* = 5.35; range: 18–52; 82% females) for GP during their first year and 126 students (*M_age_* = 21.45; *SD* = 6.17; range: 18–53; 85% females) for DEP during their second year. As these were mandatory modules, 107 participants (*M_age_* = 20.95; *SD* = 5.29; range: 18–52; 86% females) were the same for both. In other words, 65% of the students who completed the first survey also completed the second one, and 85% of the students who completed the second survey had also completed the first one. Most of the students were Italian (98% and 99%, respectively for each module).

#### 2.1.2. Research Design

The present study adopted a partially overlapping design combining longitudinal and cross-sectional elements. Data were collected in two consecutive academic modules (GP and DEP) within the same bachelor’s degree programme. Therefore, participants were recruited from the same student population across two academic years. As a result, the samples from the two modules were only partially independent: a substantial proportion of students participated in both data collections, providing repeated measurements across time, while the remaining participants contributed data to only one module.

No missing data were observed in the dataset, as all participants provided complete responses on the measures included in the analyses. Therefore, no data imputation procedures were required. Participant attrition was present across the two modules, with 107 students completing both surveys and others participating in only one module. This resulted in a partially overlapping sample, as described above.

#### 2.1.3. Procedure

We delivered two online surveys at the beginning of the first and second years. Students were invited by email, and those who provided their informed consent completed the questionnaires during the second and third weeks of lessons. Given that they had probably already attended a certain number of lessons and begun studying the modules’ contents, also reasoning about their related capacities and reactions, we presume they were able to reliably self-report their perceptions of the investigated constructs. This work is part of a longitudinal project (e.g., Raccanello et al., 2018a) approved by the Ethical Committee of the Department of Human Sciences of the University of Verona (protocol numbers 56613, 61174 and 336565, dated 17 September 2014, 18 September 2015 and 30 November 2016).

### 2.2. Measures

#### 2.2.1. Control and Value

We assessed module-related control and value. We used four items for control (*I can also learn the most difficult things about the GP/DEP module*, *I think I can do almost all the work about the GP/DEP module*, *I think I can also understand the most difficult things about the GP/DEP module*, *I know I can learn what is proposed in the GP/DEP module*) and four for value (intrinsic/unspecified value, *It is important for me to learn the contents of the GP/DEP module*; attainment value, *It is important for me to get a good grade in the GP/DEP module*; utility value, *What I learn in the GP/DEP module is useful also for other modules*, and *In my opinion, what is proposed in the GP/DEP module is useful*), adapted from other scales (Duncan & McKeachie, 2005; Lichtenfeld et al., 2012; Midgley et al., 2000; Raccanello & De Bernardi, 2010). These items were developed with experts in the field, were already available in Italian, and were used in previous projects (e.g., the Achievement Emotions Questionnaire—Elementary School project, AEQ-ES, Raccanello et al., 2019; and the Emotional Prevention and Earthquake in elementary school project, Prevenzione Emotiva e Terremoti nella scuola primaria, PrEmT, Raccanello et al., 2023). Although they reflect different facets of value (i.e., intrinsic/unspecified, attainment, and utility), they were treated as indicators of a global value appraisal, comprising both intrinsic and extrinsic components (Pekrun, 2024). This choice is consistent with the study aims and the CVT framework (Pekrun, 2006, 2024, 2025; Pekrun et al., 2023), which conceptualises value as a broad antecedent of achievement emotions, particularly when examined in interaction with control. The items were domain-specific for each module and had to be rated on a seven-point scale (1 = *Completely false for me* and 7 = *Completely true for me*). As reported in Table 2, internal consistency was good for both control (α = 0.88/0.91 and ω = 0.88/0.92, respectively, for GP/DEP) and value (α = 0.72/0.84 and ω = 0.76/0.85).

#### 2.2.2. Achievement Emotions

We measured achievement emotions by asking students to think about emotions they had felt while studying the contents of each module. Notwithstanding limitations, self-report instruments remain among the key ways to access people’s inner states (Pekrun & Bühner, 2014). We used the 30-item Achievement Emotions Adjective List (AEAL; Raccanello et al., 2022b), a relatively short scale compared to other questionnaires commonly used in this field (e.g., the Achievement Emotions Questionnaire, AEQ; Pekrun et al., 2011). This instrument is a scale measuring three positive activating (enjoyment, pride, and hope), two positive deactivating (relief and relaxation), three negative activating (anxiety, anger, and shame), and two negative deactivating emotions (hopelessness and boredom). It includes three adjectives for each emotion (e.g., *happy, satisfied, hopeful, relieved, calm, nervous, furious, ashamed, demoralised, and bored*), to be rated on a seven-point scale (1 = *Not at all* and 7 = *Very much*), assessing how much each student felt the described state. The hypothesised structure comprising 10 latent factors (corresponding to the 10 achievement emotions, with three items each) was originally tested with a sample of 2874 Swiss 10th-to-13th graders by running a confirmatory factor analysis (CFA; Raccanello et al., 2022b). The original model showed acceptable fit indexes: χ^2^(357) = 2949.28, *p* < 0.001; comparative fit index (CFI) = 0.924; Tucker–Lewis index (TLI) = 0.908; root-mean-square error of approximation (RMSEA) = 0.050; and standardised root mean residual (SRMR) = 0.046, with positive and statistically significant factor loadings (ranging from 0.49 to 0.86). In this study, as reported in Table 2, the internal consistency for each emotion was good for both GP (α/ω ranging from 0.76/0.76 to 0.88/0.89) and DEP (α/ω ranging from 0.77/0.82 to 0.92/0.93).

### 2.3. Data Analysis

We used IBM SPSS Statistics (Version 28) and Mplus (Version 8.10; Muthén & Muthén, 2017). As preliminary analyses, we tested the factorial structure of module-related control and value by conducting CFAs using maximum likelihood estimation with robust standard errors (MLR), separately for the modules of GP and DEP. Each CFA included one factor. The models are considered acceptable when the CFI and TLI are ≥0.90, and RMSEA and SRMR are ≤0.08 (Byrne, 1994; Fabrigar et al., 1999; Hu & Bentler, 1999; Kline, 2023; Marsh et al., 2005). To further investigate the factorial structure of the control and value measures, we also used an exploratory structural equation modelling (ESEM) approach. ESEM is sometimes preferable to CFA due to the likelihood of modest cross-loadings of items to other non-target factors. If the model does not take into account these cross-loadings, the fit can decline, and biassed parameters could potentially be estimated (Morin et al., 2013). For such a reason, we tested an ESEM—using the MLR estimator and the target rotation—hypothesising a two-factor structure and considering together the four items measuring control and the four items measuring value, separately for the two modules.

For achievement emotions, we verified the factorial structure of the AEAL separately for the two modules. However, considering the reduced sample sizes (i.e., 166 participants for GP and 126 for DEP), we could not test the same model described in the original article (comprising 10 latent factors; Raccanello et al., 2022b) as the ratio between observations and free parameters would have been too far from the suggested ratio (1:5; Kline, 2023). Therefore, for each module, we tested two separate CFA models—one for positive and one for negative emotions—using the MLR estimator and hypothesising a five-factor structure for each model.

We calculated descriptive statistics and intercorrelations using Pearson’s *r* (Table 2 and Table 3). According to West et al. (1995), normality assumptions are supported if skewness is lower than 2.00 and kurtosis lower than 7.00. For correlations (Table 3), we considered Cohen’s thresholds (Cohen, 1988; *r* < 0.10: very small; 0.10 ≤ *r* < 0.30: small; 0.30 ≤ *r* < 0.50: moderate; *r* ≥ 0.50: large). At an exploratory level, we examined whether the relations between control/value and each of the 10 emotions were linear and/or curvilinear, separately for each module (SM, Paragraph S2).

We then conducted 10 multiple regressions, separately for each module, with control and value as the predictors and each emotion as the outcome (Table 4 and Table 5). We checked for control–value interactions for each regression using the SPSS PROCESS macro (Model 1; Hayes, 2022). Control and value were mean-centred before the analysis to decrease potential multicollinearity effects (Aiken & West, 1991). In the case of significant interactions, we plotted simple slopes between control and the corresponding emotion at ±1 *SD* value (low level: −1 *SD*; high level: +1 *SD*; Cohen et al., 2003) and ran simple slope tests (Table 6).

To assess the adequacy of the sample size, we conducted a sensitivity power analysis for the interaction term in a multiple regression model including three predictors (control, value, and their interaction). Assuming α = 0.05 and power = 0.80, the minimum detectable effect sizes were *f*^2^ ≈ 0.049 for the GP sample (*n* = 166) and *f*^2^ ≈ 0.065 for the DEP sample (*n* = 126)—in multiple regression models, *f*^2^ represents the effect size associated with the incremental variance explained by a predictor. This indicates that the study was sufficiently powered to detect small-to-moderate interaction effects but may have been underpowered to detect smaller effects.

## 3. Results

### 3.1. Preliminary Analyses

#### 3.1.1. Control and Value

As regards control, for GP the model had acceptable CFI and SRMR, but the TLI and RMSEA were respectively lower and higher than the preferable values: χ^2^(2) = 16.883, *p* < 0.001, CFI = 0.935, TLI = 0.805, RMSEA = 0.212, SRMR = 0.035. However, acknowledging that model fit indexes are not golden rules (e.g., Marsh et al., 2004), we further examined the data searching for evidence of model misspecification. The inter-item and item-total correlations were strong (respectively, ranging from 0.526 to 0.768 and from 0.652 to 0.816). The factor loadings were positive, high, and statistically significant (ranging from 0.67 to 0.92). The proportion of explained variance (*R*^2^) was high (ranging from 0.44 to 0.85). All this considered, we found no evidence of model misspecification, leading us to conclude that the reduced sample size (*n* = 166) could be the reason for the two fit indexes (out of four) outside the thresholds. For DEP, the hypothesised single-factor model on control was confirmed, χ^2^(2) = 0.009, *p* = 0.995, CFI = 1.000, TLI = 1.000, RMSEA = 0.000, and SRMR = 0.001, with positive and statistically significant factor loadings (ranging from 0.79 to 0.95).

As for value, the hypothesised single-factor model was confirmed for both GP, χ^2^(2) = 0.149, *p* = 0.928, CFI = 1.000, TLI = 1.000, RMSEA < 0.001, SRMR = 0.006; and DEP, χ^2^(2) = 0.557, *p* = 0.757, CFI = 1.000, TLI = 1.000, RMSEA = 0.000, SRMR = 0.008, with positive and statistically significant factor loadings (ranging from 0.37 to 0.85 and from 0.55 to 0.90, respectively for the two modules).

Finally, the two-factor ESEM with the control and value as factors had good fit indexes for both GP, χ^2^(13) = 26.574, *p* = 0.014, CFI = 0.954, TLI = 0.902, RMSEA = 0.079, SRMR = 0.028; and DEP, χ^2^(13) = 22.236, *p* = 0.052, CFI = 0.977, TLI = 0.951, RMSEA = 0.075, SRMR = 0.025, supporting the structural validity of the measures. Together with the CFA findings, these results support the use of a unidimensional representation of value in the present study, despite the conceptual heterogeneity of the items.

#### 3.1.2. Achievement Emotions

For achievement emotions, the model fit indexes were acceptable for both GP (positive emotions: χ^2^(80) = 142.130, *p* < 0.001, CFI = 0.953, TLI = 0.939, RMSEA = 0.068, SRMR = 0.044; negative emotions: χ^2^(80) = 137.211, *p* < 0.001, CFI = 0.942, TLI = 0.924, RMSEA = 0.066, SRMR = 0.074) and DEP (positive emotions: χ^2^(80) = 144.350, *p* < 0.001, CFI = 0.922, TLI = 0.897, RMSEA = 0.080, SRMR = 0.057; negative emotions: χ^2^(80) = 138.926, *p* < 0.001, CFI = 0.931, TLI = 0.909, RMSEA = 0.076, SRMR = 0.067), with the only exception of TLI for positive emotions in the latter module, slightly lower than the threshold. All the factor loadings were positive and statistically significant (ranging from 0.62/0.66 to 0.94/0.93 in GP and from 0.52/0.68 to 0.91/0.94 in DEP, respectively for positive/negative emotions).

#### 3.1.3. Descriptive Statistics

We reported descriptive statistics about all the variables in Table 2. We checked skewness (range: 0.12 to 2.23) and kurtosis (range: 0.03 to 6.13) for each variable; all the values were below 2.00 (except for anger for GP) and 7.00, respectively, confirming normality assumptions.

We inserted correlations in Table 3. The correlation between control and value was positive and moderate for GP and positive and large for DEP; in both cases, it was in the same direction. For both modules, control correlated positively with positive emotions and negatively with negative emotions (except for anger and shame in GP). Value correlated positively with positive emotions (except relaxation for GP and relief for DEP) and negatively with negative emotions (except anxiety in GP).

### 3.2. Moderating Effects of Value in the Relation Between Control and Achievement Emotions

We ran 10 multiple regressions, separately by modules. The results are inserted in Table 4 for the GP module and Table 5 for the DEP module.

**Table 4:** Regression results for the General Psychology module, including main and interaction effects (final model), baseline models (without interaction), and model comparisons.

	Control		Value		Control × Value		Baseline Model			Comparison Between Baseline and Final Model		
	*b*	*p*	*b*	*p*	*b*	*p*	*R* ^2^	*F*(df)	*p*	Δ*R*^2^	Δ*F*(df)	*p*
Enjoyment	0.49	<0.001	0.38	0.002	−0.02	0.878	0.255	27.94(2,163)	<0.001	<0.001	0.02(1,162)	0.878
Pride	0.43	<0.001	0.57	<0.001	0.12	0.241	0.274	30.80(2,163)	<0.001	0.006	1.38(1,162)	0.241
Hope	0.44	<0.001	0.40	<0.001	0.13	0.138	0.274	30.77(2,163)	<0.001	0.010	2.22(1,162)	0.138
Relief	0.44	<0.001	0.18	0.149	0.19	0.063	0.149	14.25(2,163)	<0.001	0.018	3.52(1,162)	0.063
Relaxation	0.55	<0.001	−0.06	0.633	−0.15	0.159	0.181	17.98(2,163)	<0.001	0.010	2.00(1,162)	0.159
Anxiety	−0.43	<0.001	0.10	0.497	−0.07	0.562	0.091	8.17(2,163)	<0.001	0.002	0.34(1,162)	0.562
Anger	0.01	0.869	−0.45	<0.001	−0.15	0.045	0.135	12.69(2,163)	<0.001	0.021	4.08(1,162)	0.045
Shame	−0.05	0.536	−0.23	0.025	−0.17	0.033	0.038	3.18(2,163)	0.044	0.027	4.62(1,162)	0.033
Hopelessness	−0.30	<0.001	−0.34	0.002	−0.12	0.153	0.174	17.18(2,163)	<0.001	0.010	2.06(1,162)	0.153
Boredom	−0.12	0.086	−0.43	<0.001	−0.08	0.322	0.161	15.68(2,163)	<0.001	0.005	0.99(1,162)	0.322

Note. Baseline models exclude the interaction term; final models include the interaction term. *R*^2^ refers to explained variance. Δ*R*^2^ and Δ*F* indicate the change in model fit after adding the interaction. Regression coefficients (*b*) and *p*-values are reported from the final models.

**Table 5:** Regression results for the Developmental and Educational Psychology module, including main and interaction effects (final model), baseline models (without interaction), and model comparisons.

	Control		Value		Control × Value		Baseline Model			Comparison Between Baseline and Final Model		
	*b*	*p*	*b*	*p*	*b*	*p*	*R* ^2^	*F*(df)	*p*	Δ*R*^2^	Δ*F*(df)	*p*
Enjoyment	0.10	0.468	0.51	0.002	0.29	0.024	0.107	7.39(2,123)	<0.001	0.037	5.25(1,122)	0.024
Pride	0.27	0.037	0.31	0.040	0.06	0.639	0.151	10.93(2,123)	<0.001	0.002	0.22(1,122)	0.639
Hope	0.53	<0.001	−0.01	0.949	0.03	0.835	0.184	13.88(2,123)	<0.001	<0.001	0.04(1,122)	0.835
Relief	0.40	0.005	−0.15	0.365	0.23	0.079	0.063	4.15(2,123)	0.018	0.024	3.13(1,122)	0.079
Relaxation	0.76	<0.001	−0.14	0.341	0.28	0.021	0.250	20.48(2,123)	<0.001	0.032	5.45(1,122)	0.021
Anxiety	−0.47	0.002	−0.20	0.246	−0.24	0.084	0.144	10.35(2,123)	<0.001	0.021	3.04(1,122)	0.084
Anger	−0.14	0.264	−0.39	0.009	0.22	0.061	0.175	13.03(2,123)	<0.001	0.023	3.57(1,122)	0.061
Shame	−0.08	0.443	−0.40	0.001	0.09	0.376	0.186	14.09(2,123)	<0.001	0.005	0.64(1,122)	0.424
Hopelessness	−0.21	0.082	−0.29	0.048	0.09	0.424	0.147	10.56(2,123)	<0.001	0.005	0.79(1,122)	0.376
Boredom	−0.10	0.435	−0.41	0.008	−0.11	0.380	0.110	7.63(2,123)	<0.001	0.006	0.78(1,122)	0.380

Note. Baseline models exclude the interaction term; final models include the interaction term. *R*^2^ refers to explained variance. Δ*R*^2^ and Δ*F* indicate the change in model fit after adding the interaction. Regression coefficients (*b*) and *p*-values are reported from the final models.

#### 3.2.1. Positive Activating Emotions

**Figure 1:**
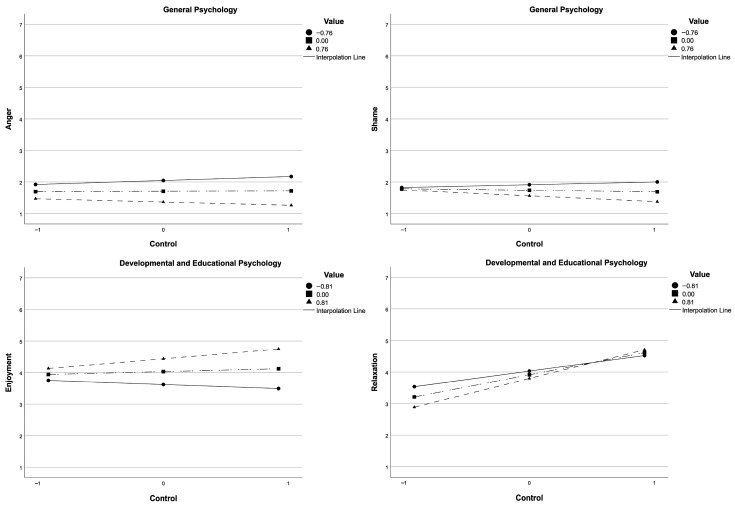
Interaction of control and value on anger and shame for General Psychology, and enjoyment and relaxation for Developmental and Educational Psychology.

Enjoyment was positively predicted by control for GP, and by value for both modules. For DEP, we also found a positive interaction between the two factors for enjoyment. Simple slope analyses (Table 6), conducted at ±1 *SD* of value, showed that the relation between control and enjoyment was positive at high levels of value but not significant at low levels. As illustrated in Figure 1, this pattern is reflected in a steeper positive slope for higher levels of value compared to lower levels. For each module, pride was positively and additively predicted by control and value. Hope was positively predicted by control for both modules and by value for GP.

**Table 6:** Results of the simple slope test for the four significant control × value interactions, separately for high (+1 *SD*) and low levels (−1 *SD*) of value (results for high value are reported before the slash and results for low value after the slash).

Module	Significant Control × Value Interaction	*b*	*t*	*p*
General Psychology	Anger	−0.10/0.12	−1.10/1.46	0.272/0.146
	Shame	0.52/0.35	4.19/3.01	<0.001/0.003
Developmental and Educational Psychology	Enjoyment	0.33/−0.14	1.98/−0.81	0.050/0.420
	Relaxation	0.99/0.54	6.22/3.33	<0.001/0.001

Control, and value, and their interaction accounted for 26% and 14% of the variance (for GP and DEP, respectively) for enjoyment, 28% and 15% for pride, and 28% and 18% for hope.

Overall, these findings provide support for H1 with respect to positive activating emotions, as control and value showed the expected positive associations. Partial support was found for H2a, as the moderating role of value emerged for enjoyment in the DEP module but not consistently across emotions and modules.

#### 3.2.2. Positive Deactivating Emotions

Only control (and not value) positively predicted relief and relaxation for both modules. For relaxation about DEP, this effect was moderated by a positive interaction between control and value. Simple slope analyses (Table 6), conducted at ±1 *SD* of value, showed that the positive relation between control and relaxation was significant at both low and high levels of value but stronger at higher levels of value. Figure 1 visually represents this interaction, with steeper slopes at higher levels of value.

The three predictors accounted for 17% and 9% of variance, respectively for GP and DEP, for relief; and 19% and 28% for relaxation.

Taken together, these results offer only partial support for H1 about positive deactivating emotions, as control—but not value—showed the expected positive associations. Evidence for H2a was limited, with a moderating effect of value observed only for relaxation in the DEP module.

#### 3.2.3. Negative Activating Emotions

For both modules, control negatively predicted anxiety. For anger and shame, control was not a significant predictor, whereas value showed a consistent negative association across modules. For anger, the two factors negatively interacted for GP. Simple slope analyses (Table 6), conducted at ±1 *SD* of value, showed that the relation between control and anger was not significant at either low or high levels of values. As shown in Figure 1, the slopes are relatively flat across levels of value, consistent with the non-significant simple slope results. For shame, there was a negative interaction for the GP. Simple slope analyses (Table 6), conducted at ±1 *SD* of value, showed that the relation between control and shame was positive and significant at both low and high levels of values. Figure 1 reflects this pattern, showing positive slopes across value levels.

For anxiety, the three predictors accounted for 9% and 17% of variance, respectively for GP and DEP; 16% and 20% for anger; and 6% and 19% for shame.

In sum, the results provide partial support for H1 with reference to negative activating emotions, as some of the expected negative associations emerged. In contrast, H2b was not supported, as the interaction effects did not consistently follow the hypothesised pattern.

#### 3.2.4. Negative Deactivating Emotions

Hopelessness was negatively predicted by control for GP, and by value for both modules. Boredom was negatively predicted by value for both modules.

For hopelessness, the three predictors accounted for 19% and 15% of variance, respectively for GP and DEP; and 17% and 12% for boredom.

Collectively, these findings provide partial support for H1 regarding negative deactivating emotions, as some of the expected negative associations emerged. No support was found for H2b, as no significant interaction effects were observed.

## 4. Discussion

We focused on the role of control and value as antecedents of 10 achievement emotions, examining their additive and multiplicative effects. We observed the same sample of university students one year apart, in relation to two mandatory psychological modules in their bachelor’s degree course. Students rated these modules as relatively high in value (see Measures/Results), and value also emerged as a salient reason underlying their emotions (see SM, Paragraph S1). Overall, the findings provided broad support for H1, although not uniformly across all emotions, whereas H2 received only partial and inconsistent support. More specifically, the additive effects of control and value were more robust than their interactive effects, which emerged only for a limited number of emotions and did not follow a uniform pattern across modules.

### 4.1. Additive Effects of Control and Value (H1)

Most of the results aligned with the CVT assumptions (Pekrun, 2006, 2024, 2025; Pekrun et al., 2023), with some exceptions. Control was related to achievement emotions, confirming H1. It positively predicted all positive activating emotions (enjoyment, pride, and hope) for each module (except enjoyment for DEP), consistently with findings about university students (Pekrun et al., 2023; Shao et al., 2020). A similar pattern emerged for the two positive deactivating emotions (relief and relaxation), positively predicted by control, for both modules. This result supports previous findings for relaxation—rarely investigated with university students—which was positively linked to perceived control but not for relief, which was negatively related in previous work (Pekrun et al., 2023). To interpret this discrepancy, it is useful to consider the two-pathway model of relief proposed by Graham et al. (2023), distinguishing between relief arising from the avoidance of a negative outcome and relief following the end of a negative state. In the present study, focusing on the studying activity rather than on evaluative outcomes, relief may have primarily reflected the second pathway, that is, the reduction in a previously experienced negative state (e.g., anxiety due to anticipated difficulty or procrastination). In such cases, higher perceived control may facilitate this reduction, leading to increased relief. Accordingly, the positive relation between control and relief observed here may reflect the predominance of this mechanism, whereas previous findings may have been more influenced by outcome-related forms of relief. Future research should explicitly distinguish between these forms to clarify their differential relations with control appraisals.

Control was significantly and negatively related only to the anticipatory outcome-focused emotions of anxiety in both modules and hopelessness in GP. For all the other negative emotions, the relations with control were not significant although the coefficients were negative (except anger in GP, for which the regression coefficient was near zero and non-significant); however, these effects should not be interpreted as statistically supported. Overall, these findings are consistent with the literature about university students (Pekrun et al., 2023; Shao et al., 2020). In addition, the relation between boredom and control was linear, supporting previous speculations about the overchallenging nature of the university tasks (Shao et al., 2020). Moreover, for anger, shame, and boredom, the mean values were quite low. Our findings tentatively suggest that when negative emotions such as anger, shame, and boredom are relatively low, students’ perceptions of control may play a less salient role in predicting them. However, the present data do not allow us to determine whether these patterns reflect stable individual differences in emotional intensity or predominance. Future research could address this issue by adopting person-centred approaches or including trait-level measures to examine whether students differ in their general emotional profiles and how these differences may influence the role of control and value appraisals. Nevertheless, keeping in mind the relatively small sample size, our findings generally confirmed the key role of perception of control in relation to all positive activating and deactivating emotions, and only some negative activating and deactivating emotions, generally supporting H1.

Also for value, the significant effects were in line with the CVT, confirming H1. Value positively predicted all positive activating emotions (except hope in DEP). However, it was not significantly related to positive deactivating emotions, although, in line with CVT, we expected the value to be positively associated with these emotions, given their positive valence and their presumed link with the perceived relevance of the task. As for negative emotions, value negatively predicted all of them except anxiety. Our results are mostly consistent with previous ones, especially for activating positive emotions and negative activity-related emotions such as anger and boredom (Pekrun et al., 2023; Shao et al., 2020), and suggest a clear pattern for positive deactivating emotions, as they are not related to value. Again, investigating students’ reasons underlying how they feel could help to understand if the apparent unrelatedness of relief and relaxation with value is due to issues concerning more specific causes salient for them that could have masked the link between this appraisal and these emotions. Being relieved or relaxed about studying could be related to future outcomes—such as one’s evaluation about one’s own learning, performance feedback, or exam grades—which however are probably not so relevant when approaching a new subject at the beginning of a new academic year, for which the formal assessment in Italy is scheduled at least three months later. The links between value and these two emotions could be stronger when measured in the proximity of the exam session. Moreover, it was not possible to determine whether these emotions, usually prospective, were focused on a temporal range included within the activity of studying or rather pertained to subsequent periods of time. As for anxiety, it could be related more to the value of failure rather than to the value types (i.e., intrinsic/unspecified, attainment, and utility value) that we investigated (Pekrun et al., 2023). Across the four categories of emotions considered (positive activating, positive deactivating, negative activating, and negative deactivating), the pattern of results varied, with stronger support for some emotional domains than others. Taken together, these findings indicate that H1 was broadly supported, especially for control and positive activating emotions, whereas support was more partial for positive deactivating and negative emotions.

### 4.2. Interactive Effects of Control and Value (H2)

As regards the exploration of the potentially interactive relation between control and value, our findings showed that value moderated relations between control and some emotions. For GP, at the beginning of the first year, this was shown for two negative activating emotions (anger and shame). However, simple slope analyses indicated that the relation between control and anger was not significant at either low or high levels of value, whereas for shame the relation was positive and significant at both levels of value. Considering previous studies, similar interactions were revealed only by Shao et al. (2020) with university students in a different context, namely a compulsory comprehensive English module for the learning setting. However, in that research the amplifying effect was stronger for low control, while in our case it was particularly relevant for high control. These differences could be related in part to some confounds due to the fact that for GP the students were at the beginning of their university course, and they had not yet received any grade as feedback about their real competence. This could explain the absence of the same interaction during the second year, in which the two emotions were predicted only by value. In other terms, it could be that the two appraisals interact to determine the two activity emotions in the studying task (i.e., when confronting the concepts and explanations about a subject, psychology, which many of them had not previously encountered at school) when appraisals about control are still not updated in line with real performance outcomes, while their role decreases after students accumulate real-world feedback.

For DEP, during the second year, a significant moderation emerged for enjoyment (consistently with Goetz et al., 2010; Putwain et al., 2018; Shao et al., 2020) and relaxation. For enjoyment, the relationship between control and emotion was positive at high levels of value, but not significant at low levels. For relaxation, the relation between control and emotion was positive at both low and high levels of value but stronger at higher levels of value. No previous study examined how control/value appraisals interacted for this emotion. This result is not completely coherent with the findings of the only study addressing the related positive deactivating emotion of contentment (Goetz et al., 2010), which is, however, outcome-focused.

To sum up, when moderation effects were observed, the pattern varied across emotions and contexts, without a consistent direction. These results provided only partial support for H2, with limited evidence for H2a (i.e., for positive emotions) and no consistent support for H2b (i.e., for negative emotions). Overall, these findings suggest that the interaction between control and value may operate differently depending on the specific emotion and context, rather than following a uniform pattern. We could argue that, for enjoyment, anger, and shame, value has a key relevance when control is high, potentially playing a role in shaping the strength of the relation between control and emotions in some contexts. When arousal is low, it would seem that, whether control and value have joint effects, the protective role of value emerges for students feeling a lack of control. Therefore, our findings indicate that the relation of control and value can be interactive, but the mechanisms underlying it could be different both for different emotions and for different contexts (in line with the domain-specificity assumptions of the CVT) and also for the same individuals—indeed, most of the sample responding to the first survey was included in the sample responding to the second one. Therefore, our research should be considered as a preliminary step in the process of accumulating empirical evidence about the additive and/or interactive relation between control and value appraisals in predicting achievement emotions, in line with the assumptions of the CVT and the few previous data on this issue (Bieg et al., 2013; Goetz et al., 2010; Lauermann et al., 2017; Putwain et al., 2018, 2021; Shao et al., 2020).

Importantly, the majority of the tested interactions were not statistically significant. This pattern suggests that, in many cases, the additive effects of control and value may be sufficient to explain achievement emotions, without requiring a multiplicative relation between the two appraisals. From a theoretical perspective, this is consistent with CVT, which does not assume that interaction effects should systematically emerge for all emotions, but rather that their occurrence may depend on specific emotional processes and contextual conditions. In particular, multiplicative effects may be more likely when both appraisals are simultaneously salient and strongly engaged, whereas in other cases emotions may be adequately explained by the independent contributions of control and value. Accordingly, the present findings highlight that the role of value as a moderator appears limited and context-dependent and that the absence of interaction effects for most emotions is itself an informative result that contributes to refining the theoretical understanding of control–value relations.

It is worth noting that the proportion of explained variance varied across emotions and modules, ranging from relatively low to moderate levels. This variability likely reflects the complexity of achievement emotions, which are influenced by multiple factors beyond control and value appraisals. While the observed *R*^2^ values are in line with previous research in educational psychology, they also indicate that additional variables may contribute to the prediction of emotions. For example, individual differences (e.g., personality traits and motivational orientations), contextual factors (e.g., teaching quality and task characteristics), and situational appraisals not captured by the present measures may play a relevant role. Furthermore, the relatively lower explained variance for some emotions (e.g., anxiety or shame for GP) may suggest that these emotions are more strongly influenced by factors such as fear of failure or social-evaluative concerns. Accordingly, future research should consider integrating additional predictors to better capture the complexity of achievement emotions.

In addition, the interaction effects observed in the present study were generally small in magnitude, which is consistent with previous literature showing that moderation effects in non-experimental designs are often modest and difficult to detect (McClelland & Judd, 1993). Accordingly, the available sample size may have limited the statistical power to detect weaker interaction effects, potentially contributing to the inconsistency of findings across emotions and modules. This aspect should be considered when interpreting the results related to H2, which were exploratory in nature.

Finally, given the relatively large number of interaction tests conducted (10 emotions × 2 modules), the possibility of a Type I error cannot be excluded. Supplementary analyses applying a false discovery rate correction (Benjamini–Hochberg), reported in the SM (Paragraph S3), further suggest that these interaction effects should be interpreted with caution. Future research should consider applying corrections for multiple comparisons and replicating these findings in larger samples.

### 4.3. Applied Implications

This study suffers from some limitations. On the whole, our results support the basic idea that changing both control and value perceptions could impact students’ achievement emotions and, in light of the links between achievement emotions and performance, also their learning outcomes. On the one hand, it could be worth implementing a series of activities and practices with students aiming at ameliorating their feelings of control over a task. Control can be promoted by giving students the possibility to reflect on their control over tasks, by reframing their attributional beliefs (e.g., also through evidence-based interventions; see Hamm et al., 2014 for an example of an evidence-based attributional retraining treatment), or by providing them with direct or indirect mastery experiences and supporting them through external positive feedback (Bandura, 2018) and teachers’ enthusiasm (Moè et al., 2021). Our data indicate that at the university level increases in perceived control could have, in turn, the effect of improving many of the positive emotions that can be felt while learning and could also have beneficial effects in terms of diminishing anxiety, frequently associated with low achievement (Pekrun et al., 2023). However, we acknowledge that self-efficacy and similar beliefs could have detrimental effects in case they are not realistic (Talsma et al., 2019). Therefore, it is worth working on students’ control appraisals, but with the awareness that in some cases it would be better not to foster an unrealistic view about one’s capacities.

On the other hand, the previous literature indicated, also through evidence-based procedures, that some programmes influence not only students’ control but also the value that they attribute to a task (Eccles & Wigfield, 2023). This happens for example when the environment supports students’ needs respecting the rhythm of their learning, giving rationales underlying instructions and rules, proposing different options, favouring mastery experiences, and using an informative language (Reeve, 2009, 2016), also through the adoption of specific programmes (e.g., Cheon et al., 2018). Most of these programmes tried to improve utility value, to foster cascade effects in students’ motivation and performance (Harackiewicz & Priniski, 2018). While intervention programmes aiming at improving the control and the value of studying could be useful in general for students with all levels of capacities, it is worth considering that they could be differentially efficacious for students perceiving themselves at different levels of competence. Indeed, some of the interactions that we detected suggest that intervention programmes could have differential effects for different emotions. Considering positive emotions, if we focus on enjoyment, such programmes could be particularly useful for those students perceiving themselves as very competent. If we consider relaxation, we could formulate opposite expectations, with particular relevance of such programmes for students with low control. Again, our results about the moderation of control and value for anger and shame suggest that value may play a role in modulating emotional experiences for students’ well-being and performance, especially for those students who perceive themselves as particularly competent. Having in mind the possible negative effects of these two emotions for learning (Pekrun et al., 2023), in planning interventions to ameliorate students’ perception of value towards a subject, particular attention should be paid to taking into account their perceived competence. Nevertheless, given that our findings about interactions in part differed from previous research, we should be cautious before generalising them to other contexts.

### 4.4. Limitations and Directions for Future Research

This study suffers from some limitations. First, we initially proposed the survey to all the students enrolled in the first year of the bachelor’s degree course in psychological sciences. We do not know the reasons underlying their decision to participate or not. The students accepting participation could have been more motivated or engaged towards their studies, and so the sample may not be representative at the course level. Moreover, the total number of students enrolled each year in the degree course under investigation was 225; therefore, the size of our two samples was smaller than that number, but still relatively representative of the population (74% of the population for the first year and 56% for the second year). In any case, the sample size was relatively modest, particularly with respect to detecting interaction effects in multiple regression analyses, which typically require larger samples (McClelland & Judd, 1993). This may have reduced statistical power and contributed to the limited and inconsistent evidence for interaction effects.

Second, the students were involved at the beginning of their academic year, so they could still have been in their initial stages in the development of their control and value perceptions about the subjects and the topics, especially for the first year; future research should gather measures at more time points to monitor this aspect. In addition, given that the selected modules were very relevant for students’ degree course, both control and value had relatively high scores. Future research could aim to involve larger and more representative samples, paying more attention to recruitment issues and balancing modules according to their relevance. Moreover, another limitation of the present study concerns the operationalisation of value as a unidimensional construct. Although the items covered different facets (e.g., intrinsic, attainment, and utility value), they were aggregated into a single factor. Future research should disentangle these components to examine whether they differentially interact with control in predicting specific achievement emotions.

Third, in the sample there was a prevalence of females. While in the future it would be worth exploring these issues with more balanced samples, this is a typical characteristic in the composition of the groups of students enrolled in Italian psychology university courses (Morana & Sagramora, 2019).

Fourth, the dataset included both a cross-sectional component (independent observations from students participating in only one module) and a longitudinal component (repeated observations from the same individuals across modules). For the purposes of the present study, analyses were conducted separately for each module to examine the relations between control, value, and achievement emotions within each academic context. However, given the partial overlap between samples, the two datasets cannot be considered fully independent, and comparisons across modules should be interpreted with caution. In addition, the data concerning each module were correlational in nature. Future studies could examine the relations between individual antecedents of emotions and emotions considering more than one time point, e.g., at the beginning, middle, and end of a module, to disentangle reciprocal feedback loops as postulated by the CVT. In addition, we did not consider the relation of control and value appraisals and achievement emotions with achievement. In the future, it could be interesting to investigate how both appraisals and emotions are related to later performance through a longitudinal design.

Fifth, we based our research on self-report instruments and mainly on closed questions, which are still among the most relevant ways to get insight into people’s inner world (Pekrun & Bühner, 2014). These data could be triangulated by both coding responses to open questions about students’ emotions and adding physiological or observational measures. Coding responses to open questions could also shed light on students’ range of reasons for feeling specific emotions in specific time periods and settings. Moreover, while our instructions solicited the students to report the achievement emotions that they felt while studying—which were probably concurrent, activity-focused emotions—we could not exclude that they also referred to temporal frames different from the present. For example, this could have happened for emotions typically focused retrospectively on the past, like relief, or prospectively on the future, like anxiety. Future research could add questions to investigate explicitly on which object type and temporal relation the measured emotions are focused on.

Sixth, another limitation concerns the proportion of explained variance. Although control and value significantly predicted several emotions, the overall amount of variance explained was relatively modest for some outcomes. This suggests that other relevant predictors, not included in the present study, may contribute to students’ emotional experiences.

Finally, most of the studies investigating the interaction between control and value focused on the learning setting. Future research could investigate if the same effects generalise to other settings, such as exams. Given that some achievement emotions result as more salient in evaluative versus non-evaluative settings (Raccanello et al., 2013), in line with CVT assumptions about their changing rates according to individual dispositions (Frenzel et al., 2007a; Pelch, 2018) or cultural contexts (Frenzel et al., 2007b), it could be worth testing the universality of the interaction between different appraisals and emotions (Pekrun, 2006, 2024).

## 5. Conclusions

This study provides evidence that control and value appraisals are relevant for predicting a broad range of positive and negative, activating and deactivating achievement emotions within the specific context of university students’ studying activities in two psychological modules. Moreover, it offers partial support for the role of value as a moderator, as interaction effects emerged only for some emotions. Taken together, the findings suggest that the additive effects of control and value are relatively robust, whereas their interactive effects appear more context-dependent and less stable across emotional outcomes. In this sense, the study contributes to refining the assumptions of CVT by highlighting the need to further investigate when and for which emotions multiplicative effects are more likely to emerge.

From an applied perspective, the results indicate that interventions aimed at enhancing students’ perceptions of control and value may foster more adaptive emotional experiences, although their effectiveness may vary depending on the specific emotional processes involved. Notwithstanding the study’s limitations, these findings extend the investigation of CVT assumptions and underscore the importance of future research examining a wider range of emotions and contexts, as well as employing larger samples and more robust designs to clarify the role of control–value interactions.

## Figures and Tables

**Table 1:** Four quadrants derived from the combination of achievement emotions differing in valence (positive and negative) and physiological arousal (activating and deactivating).

	Activating Emotions	Deactivating Emotions
**Positive Emotions**	Enjoyment; Pride; Hope	Relief; Relaxation
**Negative Emotions**	Anxiety; Anger; Shame	Hopelessness; Boredom

**Table 2:** Descriptive statistics for study variables by module (General Psychology, before the slash, and Developmental and Educational Psychology, after the slash).

	Range	*M*	*SD*	Skewness	Kurtosis	Cronbach’s α	McDonald’s ω
Control	1–7/1–7	5.19/5.44	1.02/0.92	−0.13/−0.26	−0.67/−0.58	0.88/0.91	0.88/0.92
Value	1–7/1–7	6.18/5.92	0.76/0.81	−1.04/−0.48	0.53/−0.49	0.72/0.84	0.76/0.85
Enjoyment	1–7/1–7	4.53/4.16	1.30/1.18	−0.17/0.22	−0.61/−0.41	0.88/0.87	0.89/0.87
Pride	1–7/1–7	4.73/4.50	1.35/1.12	−0.22/0.12	−0.36/−0.50	0.86/0.77	0.88/0.82
Hope	1–7/1–7	4.89/4.88	1.18/1.12	−0.12/−0.08	−0.58/−0.29	0.79/0.85	0.80/0.85
Relief	1–7/1–7	3.35/3.56	1.28/1.18	0.53/0.47	0.17/0.22	0.86/0.84	0.86/0.85
Relaxation	1–7/1–7	3.89/4.04	1.32/1.21	0.13/0.15	−0.40/−0.27	0.88/0.84	0.88/0.85
Anxiety	1–7/1–7	3.32/3.01	1.38/1.30	0.18/0.44	−0.41/−0.41	0.80/0.82	0.81/0.82
Anger	1–7/1–7	1.67/1.84	0.90/1.12	2.23/1.47	6.13/1.41	0.87/0.92	0.88/0.93
Shame	1–7/1–7	1.70/1.64	0.96/0.92	1.44/1.82	1.28/3.35	0.85/0.87	0.86/0.87
Hopelessness	1–7/1–7	2.14/2.27	1.09/1.06	1.17/0.92	1.58/0.50	0.86/0.81	0.86/0.82
Boredom	1–7/1–7	2.20/2.35	0.96/1.10	0.84/0.82	0.79/−0.03	0.76/0.82	0.76/0.82

**Table 3:** Bivariate correlations for study variables (General Psychology, below the diagonal, and Developmental and Educational Psychology, above the diagonal).

Variable	1	2	3	4	5	6	7	8	9	10	11	12
1. Control	-	0.606 ***	0.245 **	0.350 ***	0.429 ***	0.217 *	0.483 ***	−0.375 ***	−0.319 ***	−0.308 ***	−0.330 ***	−0.251 **
2. Value	0.327 ***	-	0.322 ***	0.346 ***	0.251 **	0.030	0.190 *	−0.273 **	−0.408 ***	−0.427 ***	−0.354 **	−0.325 ***
3. Enjoyment	0.459 ***	0.349 ***	-	0.743 ***	0.563 ***	0.524 ***	0.575 ***	−0.208 *	0.016	0.038	−0.071	−0.151
4. Pride	0.428 ***	0.425 ***	0.792 ***	-	0.554 ***	0.527 ***	0.457 ***	−0.177 *	−0.045	−0.058	−0.179 *	−0.213 *
5. Hope	0.462 ***	0.384 ***	0.683 ***	0.697 ***	-	0.478 ***	0.516 ***	−0.185 *	−0.154	−0.104	−0.205 *	−0.180 *
6. Relief	0.372 ***	0.218 **	0.685 ***	0.672 ***	0.618 ***	-	0.720 ***	−0.284 **	0.096	0.173	−0.073	−0.086
7. Relaxation	0.424 ***	0.108	0.626 ***	0.459 ***	0.565 ***	0.664 ***	-	−0.522 ***	−0.070	−0.014	−0.215 *	−0.170
8. Anxiety	−0.297 ***	−0.048	−0.225 **	−0.191 *	0.240 **	−0.346 ***	−0.523 ***	-	0.450 ***	0.463 ***	0.639 ***	0.541 ***
9. Anger	−0.097	−0.366 ***	−0.290 ***	−0.284 ***	−0.282 ***	−0.128	−0.218 **	0.366 ***	-	0.757 ***	0.719 ***	0.714 ***
10. Shame	−0.094	−0.191 *	−0.078	−0.127	−0.113	0.001	−0.088	0.450 ***	0.519 ***	-	0.695 ***	0.610 ***
11. Hopelessness	−0.352 ***	−0.327 ***	−0.362 ***	−0.441 ***	−0.411 ***	−0.376 ***	−0.383 ***	0.604 ***	0.597 ***	0.474 ***	-	0.668 ***
12. Boredom	−0.237 **	−0.384 ***	−0.426 ***	−0.385 ***	−0.316 ***	−0.297 ***	−0.255 ***	0.427 ***	0.591 ***	0.396 ***	0.654 ***	-

Note. * *p* < 0.05. ** *p* < 0.01. *** *p* < 0.001.

## Data Availability

The original data presented in the study are openly available in Open Science Framework at https://osf.io/w2raz/overview.

## Supplementary Materials

The following supporting information can be downloaded at: https://www.mdpi.com/article/10.3390/bs16050791/s1. Paragraph S1: Exploration of Students’ Reasons for Their Module-Related Emotions; Paragraph S2: Exploratory Curve Estimation; Paragraph S3: Correction for Multiple Comparisons of Interaction Effects; Table S1: Curve estimation of the linear and quadratic relations between control and achievement emotions, separately by module (values about General Psychology are reported before the slash, and values about Developmental and Educational Psychology after the slash); Table S2: Curve estimation of the linear and quadratic relations between value and achievement emotions, separately by module (values about General Psychology are reported before the slash, and values about Developmental and Educational Psychology after the slash); Table S3: Benjamini–Hochberg (BH) correction for the interaction effects for General Psychology; Table S4: Benjamini–Hochberg (BH) correction for the interaction effects for Developmental and Educational Psychology; Figure S1: Scatter plots of the linear and quadratic relations between value and enjoyment for the module of Developmental and Educational Psychology, separately for (a) low (−1 *SD*), (b) mean (0 *SD*), and (c) high (+1 *SD*) control.

## Abbreviations

The following abbreviations are used in this manuscript:

AEAL	Achievement Emotions Adjective List
CVT	Control–value theory
DEP	Developmental and Educational Psychology
GP	General Psychology
SM	Supplementary Materials
