# Supplementary material for: How Perceived Control and Task Value Relate to Achievement Emotions in Academic Study Settings
Source: Behav Sci (Basel). 2026 May 16;16(5):791. doi: 10.3390/bs16050791 (PMC13203922; doi:10.3390/bs16050791)
Supplement: Supplementary file 1 [file behavsci-16-00791-s001.zip › behavsci-4250418-supplementary.pdf]

behavioral sciences

MDPI

Supplementary Materials

How Perceived Control and Task Value Relate to Achievement Emotions in Academic Study Settings

Daniela Raccanello [1,2,*], Giada Vicentini [1,2] and David W. Putwain [2]

[1] Department of Human Sciences, University of Verona, 37129 Verona, Italy; giada.vicentini@univr.it
[2] School of Education, Liverpool John Moores University, Liverpool L1 9DE, UK; d.w.putwain@ljmu.ac.uk
* Correspondence: daniela.raccanello@univr.it

Paragraph S1

S1 Exploration of Students' Reasons for Their Module-Related Emotions

S1.1. Aim

As a preliminary, we checked whether the students perceived the two modules (i.e., General Psychology, GP, and Developmental and Educational Psychology, DEP) as valuable activities, through an open question investigating the reasons for their module-related emotions. In this case, our aim was to further explore students' representation of value for the two modules, investigating whether value emerged as a salient reason underlying their module-related emotions. We expected the students to frequently mention value (in terms of intrinsic, attainment, and/or utility value) as one of the reasons underlying the emotions that they felt in relation to the two modules on which we focused. Mentioning this construct could have been an indicator of the salience of value in their representation of each module (Putwain et al., 2018), confirming the relevance of the two modules under investigation for the involved students.

S1.2. Method

S1.2.1. Participants and Procedure

See the description in the manuscript.

S1.2.2. Measures

We asked the students to respond to an open question (adapted from Raccanello et al., 2018b) about their module-related emotions and underlying reasons (i.e., *How do you feel when you study for the GP/DEP module? Why?*). Salience of module-related value was operationalised by coding the responses for the reference to at least one value type: intrinsic value (e.g., *Eagerness to discover* […] *it is really such a wide subject that you feel immersed in a world bigger than you. As being in the middle of New York without having visited it yet* / *I feel curious because I learn new things*, respectively for the two modules), attainment value (e.g., *I'm afraid not being up to the exam* / *I feel worried about achieving my best*), or utility value (e.g., *These are modules and knowledge necessary for the future I imagine* / *I think that* [the contents] *are very useful for my future job*). For each participant, the score was 0 if there was no mention of any kind of value; the score was 1 if they mentioned at least one value type. A first judge coded all the responses; a second judge coded 30% of the responses. Inter-rater reliability was very good, given that the percentage agreement between the two judges for

the two modules was, respectively, 100% and 97%. Discrepancies were solved through discussion between the judges.

S1.3. Results

We examined students' responses about the reasons for their emotions, as gathered using the open questions. We calculated the percentage of responses referring to each module's value (unspecified, attainment, and utility value).

For GP, we found that most of the students (87%) spontaneously reported value as one of the reasons for their emotions. Again, one year later, for DEP, most of the students (82%) cited value as one of the reasons for how they felt about the module in question. Therefore, these data suggested that the students recognized personal value as a salient antecedent of their module-related emotions.

S1.4. Discussion

Preliminarily, we used open questions to gather information about the salience of value as one of the reasons underlying how they felt in relation to the two modules. The exam of their responses highlighted that value—in its different nuances, i.e., in terms of intrinsic, attainment, or utility value—is a dimension salient as one determinant of emotions within students' representations of both modules (Putwain et al., 2018).

These qualitative data indicate that value is salient in students' representations of both modules, highlighting its role as an antecedent of their emotions. Importantly, they speak to the salience of value rather than its level, as the spontaneous mention of value does not in itself imply high evaluations. The level of value is instead supported by the quantitative ratings, which showed relatively high mean scores for both GP and DEP. Taken together, these findings provide complementary evidence, with qualitative data capturing the prominence of value in students' meaning-making and quantitative data indicating relatively high value ratings.

Paragraph S2

S2 Exploratory Curve Estimation

At an exploratory level, we examined whether relations between control or value and each of the 10 achievement emotions were linear or curvilinear (i.e., quadratic), separately for the two modules (see Table S1 and Table S2). About control (Table S1), there was a significant linear relation in all the cases except for control and anger, and for control and shame for GP. About value (Table S2), there was a significant linear relation in all the cases except, for GP, for value and relaxation, and for value and anxiety, and, for DEP, for value and relief. The results also showed, for GP, a significant quadratic relation between control and pride, and between value and relaxation, and for DEP a significant quadratic relation between value and enjoyment, anger, and shame.

The regression analyses revealed a significant positive interaction only between control and value in predicting enjoyment, for the module of DEP. Therefore, we ran again the curve estimation analysis for enjoyment. We tested the linear and quadratic relation between value and enjoyment, differentiating the groups of students with low (−1 *SD*; n = 22), mean (0 *SD*; n = 84), and high (+1 *SD*; n = 30) control (see Figure S1a, S1b, and S1c, respectively). The linear relation was significant at mean (b = 0.53, p = 0.002) but not at low (b = −0.33, p = 0.259) or high (b = 1.25, p = 0.088) levels of control. As regards the curvilinear relation, it was significant only for participants with low (b = 0.80, p = 0.016) levels of control, but not for those with mean (b = 0.11, p = 0.630) or high (b = −1.10, p = 0.508) levels.

Table S1. Curve estimation of the linear and quadratic relations between control and achievement emotions, separately by module (values about General Psychology are reported before the slash, and values about Developmental and Educational Psychology after the slash).

	Linear		Quadratic	
	b	***p***	***b***	***p***
Enjoyment	0.58/0.31	< 0.001/0.006	−0.11/0.05	0.151/0.609
Pride	0.56/0.43	< 0.001/< 0.001	−0.16/−0.04	0.040/0.663
Hope	0.53/0.52	< 0.001/< 0.001	−0.09/−0.16	0.205/0.087
Relief	0.47/0.28	< 0.001/0.015	0.03/−0.05	0.711/0.652
Relaxation	0.55/0.64	< 0.001/< 0.001	−0.02/0.05	0.813/0.606
Anxiety	−0.40/−0.53	< 0.001/< 0.001	−0.16/−0.14	0.079/0.220
Anger	−0.09/−0.39	0.215/< 0.001	0.01/0.04	0.924/0.703
Shame	−0.09/−0.31	0.229/< 0.001	−0.06/−0.09	0.332/0.290
Hopelessness	−0.37/−0.38	< 0.001/< 0.001	0.01/−0.03	0.899/0.759
Boredom	−0.22/−0.30	0.002/0.005	−0.08/0.01	0.178/0.942

Table S2. Curve estimation of the linear and quadratic relations between value and achievement emotions, separately by module (values about General Psychology are reported before the slash, and values about Developmental and Educational Psychology after the slash).

	Linear		Quadratic	
	b	***p***	***b***	***p***
Enjoyment	0.59/0.47	< 0.001/< 0.001	0.13/0.40	0.364/0.003
Pride	0.75/0.48	< 0.001/< 0.001	−0.12/0.19	0.392/0.137
Hope	0.59/0.35	< 0.001/0.005	0.13/0.07	0.280/0.600
Relief	0.37/0.04	0.005/0.738	−0.14/0.21	0.337/0.159
Relaxation	0.19/0.29	0.165/0.034	0.29/0.16	0.049/0.278
Anxiety	−0.09/−0.44	0.535/0.002	−0.01/−0.04	0.980/0.809
Anger	−0.43/−0.57	< 0.001/< 0.001	0.06/0.36	0.549/0.003
Shame	−0.24/−0.49	0.014/< 0.001	0.04/0.20	0.743/0.048
Hopelessness	−0.47/−0.46	< 0.001/< 0.001	0.22/0.10	0.056/0.410
Boredom	−0.48/−0.44	< 0.001/< 0.001	0.06/−0.02	0.558/0.896

Figure S1. Scatter plots of the linear and quadratic relations between value and enjoyment for the module of Developmental and Educational Psychology, separately for (a) low (-1 *SD*), (b) mean (0 *SD*), and (c) high (+1 *SD*) control.

Figure S1a

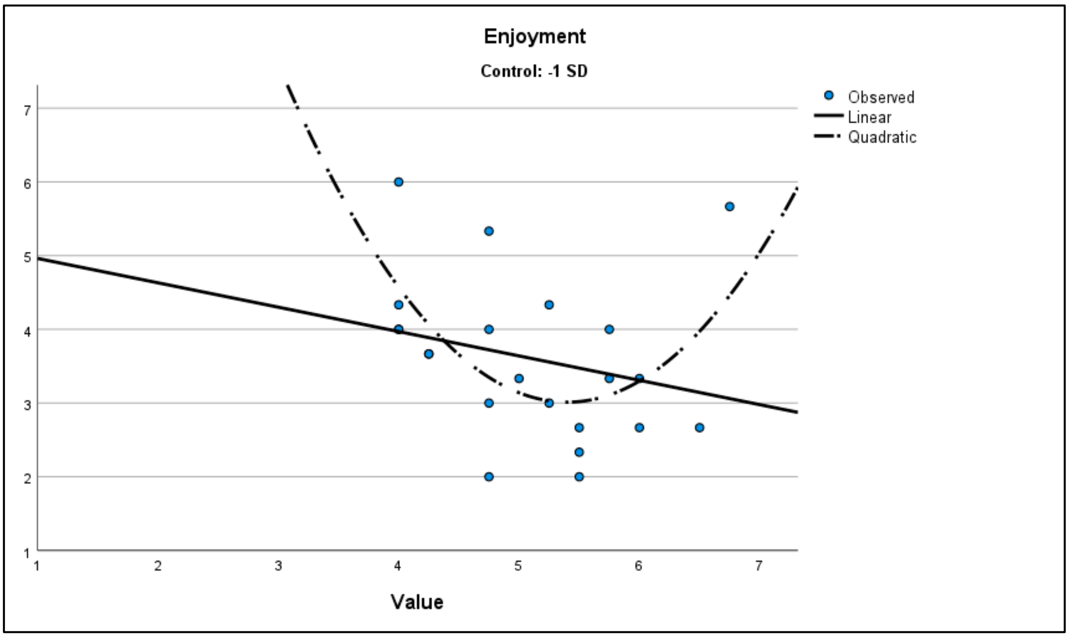

Figure S1b

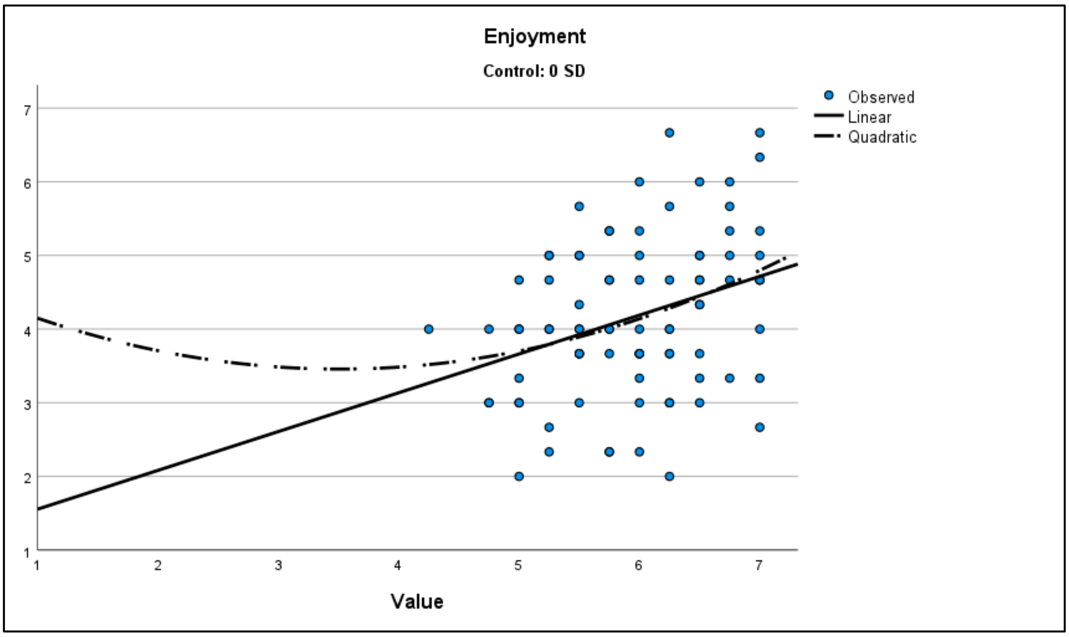

Figure S1c

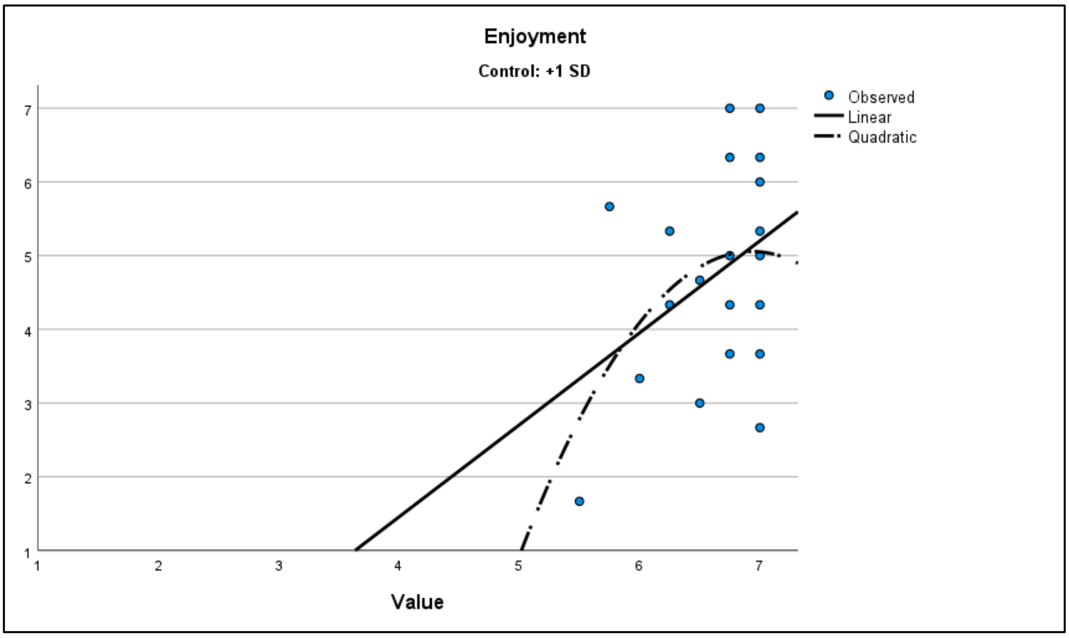

Paragraph S3

S3 Correction for Multiple Comparisons of Interaction Effects

To account for the issue of multiple comparisons, a false discovery rate (FDR) correction was applied to the interaction effects using the Benjamini–Hochberg procedure, separately for each module ($m = 10$ tests per module). Adjusted p-values are reported in Table S3 for GP and Table S4 for DEP. After correction, none of the interaction effects remained statistically significant at $\alpha = 0.05$.

Table S3. Benjamini–Hochberg (BH) correction for the interaction effects for General Psychology.

	**Interaction *b***	**Uncorrected *p***	**BH-Adjusted *p***	**Significant After BH**
Enjoyment	−0.02	0.878	0.878	No
Pride	0.12	0.241	0.344	No
Hope	0.13	0.138	0.265	No
Relief	0.19	0.063	0.210	No
Relaxation	−0.15	0.159	0.265	No
Anxiety	−0.07	0.562	0.624	No
Anger	−0.15	0.045	0.210	No
Shame	−0.17	0.033	0.210	No
Hopelessness	−0.12	0.153	0.265	No
Boredom	−0.08	0.322	0.403	No

Note. Benjamini–Hochberg correction was applied separately within each module across the 10 interaction tests. Regression coefficients (b) are reported from the final models. BH-adjusted p-values reflect false discovery rate control at $\alpha = 0.05$.

Table S4. Benjamini–Hochberg (BH) correction for the interaction effects for Developmental and Educational Psychology.

	Interaction *b*	Uncorrected *p*	BH-Adjusted *p*	Significant After BH
Enjoyment	0.29	0.024	0.120	No
Pride	0.06	0.639	0.710	No
Hope	0.03	0.835	0.835	No
Relief	0.23	0.079	0.168	No
Relaxation	0.28	0.021	0.120	No
Anxiety	−0.24	0.084	0.168	No
Anger	0.22	0.061	0.168	No
Shame	0.09	0.376	0.530	No
Hopelessness	0.09	0.424	0.530	No
Boredom	−0.11	0.380	0.530	No

Note. Benjamini–Hochberg correction was applied separately within each module across the 10 interaction tests. Regression coefficients (b) are reported from the final models. BH-adjusted p-values reflect false discovery rate control at $\alpha = 0.05$.

References

Putwain, D. W., Pekrun, R., Nicholson, L. J., Symes, W., Becker, S., & Marsh, H. W. (2018). Control-value appraisals, enjoyment, and boredom in mathematics: A longitudinal latent interaction analysis. *American Educational Research Journal*, *55*(6), 1339–1368. https://doi.org/10.3102/0002831218786689.

Raccanello, D., Hall, R., & Burro, R. (2018b). Salience of primary and secondary school students' achievement emotions and perceived antecedents: Interviews on literacy and mathematics domains. *Learning and Individual Differences*, *65*, 65–79. https://doi.org/10.1016/j.lindif.2018.05.015.

Disclaimer/Publisher's Note: The statements, opinions and data contained in all publications are solely those of the individual author(s) and contributor(s) and not of MDPI and/or the editor(s). MDPI and/or the editor(s) disclaim responsibility for any injury to people or property resulting from any ideas, methods, instructions or products referred to in the content.
